# Analysing complex *Triticeae* genomes – concepts and strategies

**DOI:** 10.1186/1746-4811-9-35

**Published:** 2013-09-06

**Authors:** Manuel Spannagl, Mihaela M Martis, Matthias Pfeifer, Thomas Nussbaumer, Klaus FX Mayer

**Affiliations:** 1 MIPS/IBIS, Helmholtz Center Munich, National Research Center for Environment and Health, Ingolstaedter Landstr. 1, Neuherberg, Germany

**Keywords:** *Triticeae* genomes, Grass genomes, Wheat genome, Barley genome, GenomeZipper, Genome analysis

## Abstract

The genomic sequences of many important *Triticeae* crop species are hard to assemble and analyse due to their large genome sizes, (in part) polyploid genomes and high repeat content. Recently, the draft genomes of barley and bread wheat were reported thanks to cost-efficient and fast NGS technologies. The genome of barley is estimated to be 5 Gb in size whereas the genome of bread wheat accounts for 17 Gb and harbours an allo-hexaploid genome. Direct assembly of the sequence reads and access to the gene content is hampered by the repeat content. As a consequence, novel strategies and data analysis concepts had to be developed to provide much-needed whole genome sequence surveys and access to the gene repertoires. Here we describe some analytical strategies that now enable structuring of massive NGS data generated and pave the way towards structured and ordered sequence data and gene order. Specifically we report on the GenomeZipper, a synteny driven approach to order and structure NGS survey sequences of grass genomes that lack a physical map. In addition, to access and analyse the gene repertoire of allo-hexaploid bread wheat from the raw sequence reads, a reference-guided approach was developed utilizing representative genes from rice, *Brachypodium distachyon*, sorghum and barley. Stringent sub-assembly on the reference genes prevented collapsing of homeologous wheat genes and allowed to estimate gene retention rate and determine gene family sizes. Genomic sequences from the wheat sub-genome progenitors enabled to discriminate a large number of sub-assemblies between the wheat A, B or D sub-genome using machine learning algorithms. Many of the concepts outlined here can readily be applied to other complex plant and non-plant genomes.

## Review

### Introduction

The *Triticeae* tribe comprises some of the most economically important crops including bread wheat, barley and rye. Bread wheat ranked third in world crop production with 681 million tons in 2011 [1], making it an indispensable source for our everyday diet. Domestication history of *Triticeae* dates back several thousand years. They consequently have a complex genetic history [2].

The genomes of many *Triticeae* species including wheat and barley appear to be extremely challenging to assemble and analyse due to their genome size, high repeat content, complex transposable element structure and, in part, polyploid genome [3,4].

With an estimated genome size of ~5 Gb the barley genome is significantly larger than the human genome, however exceeded by the bread wheat genome with ~17 Gb. Bread wheat contains an allo-hexaploid genome with three sub-genomes, namely the A, B and D sub-genome. It has been speculated that the bread wheat genome originated from hybridization between cultivated tetraploid emmer wheat (AABB) and diploid goat grass (DD) about 8000 years ago [5].

Complementing the genome size, many *Triticeae* genomes show a very high degree of repetitive elements (~80% in bread wheat [6]). These repetitive stretches can span several 100kbs and have a complex architecture and composition. Consequently assembly of long scaffolds or even whole chromosome sequences from NGS survey sequences using current technology is still an open problem [Review [7]]. Although synteny is pronounced in grasses and in general the gene order appears to be well conserved [8], repeat and TE activities as well as structural rearrangements contribute to the formation of pseudogenes, gene fragments and changes in local gene order [4].

With the availability of economic and rapid NGS technologies whole-genome sequence surveys of many grass genomes including a number of *Triticeae* species were generated recently [9,10]. While the direct assembly of reads into pseudo-chromosomes or scaffolds is hampered by the genomes’ repetitiveness and size, the gene inventory, gene order and chromosomal positioning of genetic elements such as genes and markers is of high interest not only for breeders but also helps to shed light on the evolutionary history of the respective plants and the *Triticeae* in general.

Consequently, numerous novel strategies and concepts were developed over the last few years to order [11,12], analyse [9] and compare complex *Triticeae* genomes even in the light of the challenges and limitations described. Here, we highlight a few of these concepts that were applied to analyse the recently published genome sequences of barley [10] and bread wheat [9] and describe the methodology used in more detail.

Many of the concepts and strategies described and discussed here are not restricted to the *Triticeae* but can be applied to other complex grass and plant genomes that have not been sequenced and analysed so far due to their genome size and/or polyploid nature.

### A strategy for the comprehensive analysis of polyploid genomes. an ortholome approach for the analysis of hexaploid bread wheat

**Figure 1:**
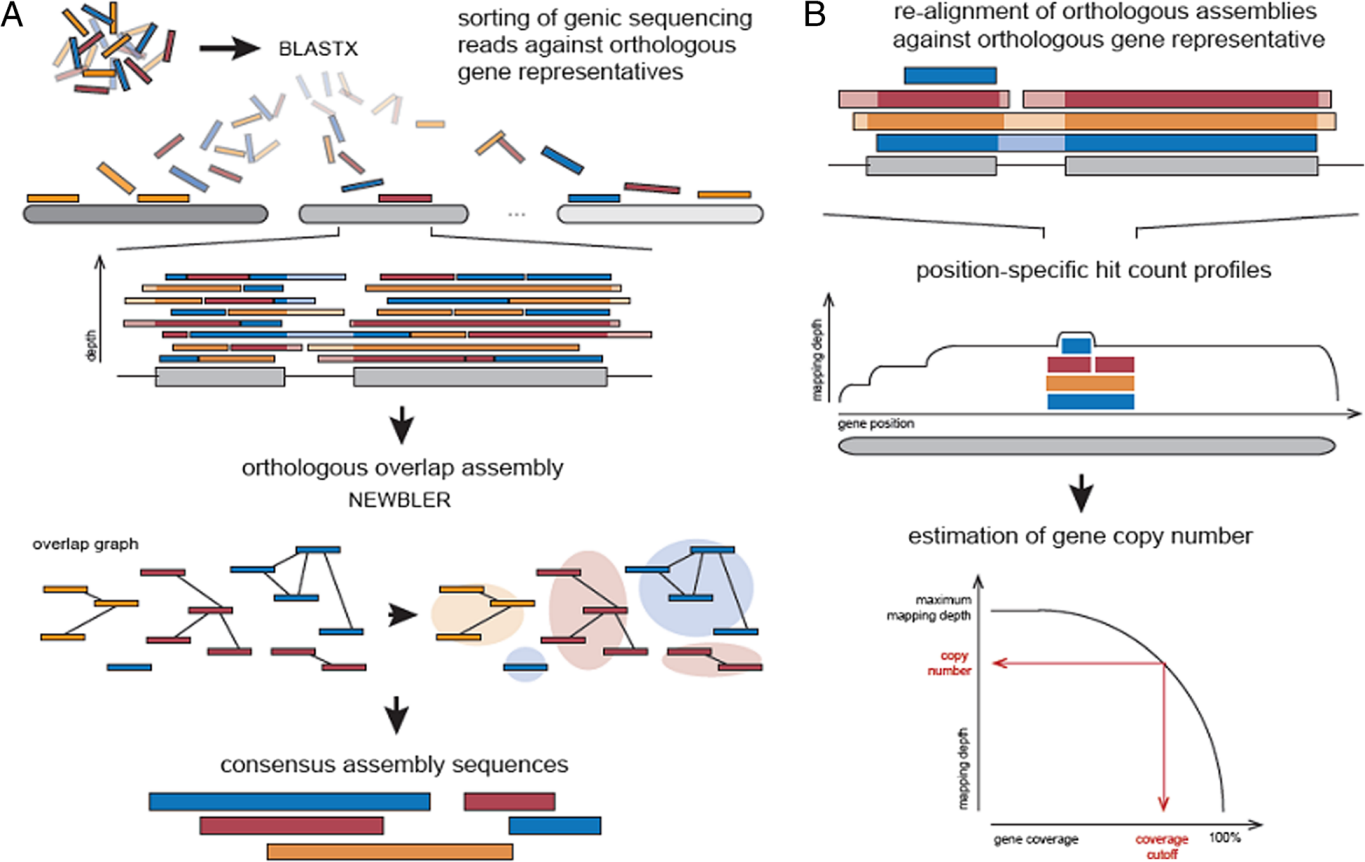
**Schematic representation of the orthologous group assembly workflow and the protocol for the estimation of gene copy number.** Grey boxes represent the protein sequence of orthologous group representative, whereas lines connecting boxes depict exon boundaries. Coloured boxes visualize sequencing reads and assembled sequences, respectively. The colour code groups sequences that originate from the same genome and light colouring visualize non-coding regions. **A** The orthologous assembly algorithm sorts the raw sequencing reads to corresponding orthologous gene representatives based on sequence similarity (BLASTX). Then, each sequence bin is separately assembled using NEWBLER, an overlap graph assembler which identifies overlapping sequence reads first and then creates a consensus assembly sequence based on the overlap graph. **B** For estimating the gene copy number the orthologous assembly sequences were re-aligned to the corresponding template (BLASTX) and, thus, ordered along its protein sequence. The alignments are transferred into a position-specific hit count profile that counts the number of distinct sub-assemblies mapped to each amino acid of the template protein. Based on the cumulative coverage distribution of the hit-count profile, the final gene copy number is determined as the maximum number of distinct sub-assemblies covering a defined proportion of the template gene.

The orthologous group assembly (OA) is a strategy which aims to identify the gene repertoire of polyploid genomes based on low- and medium coverage, long-read (454) whole genome shotgun data. This approach was applied for the comprehensive sequence analysis of hexaploid bread wheat [9] and facilitated the identification of 94,000-96,000 wheat genes. Due to a stringent assembly protocol, rare sequence polymorphisms are sufficient in order to maintain and distinguish distinct copies of homeologous genes, which might be collapsed by a brute-force *de novo* assembly. In contrast to traditional assembly approaches, the orthologous group assembly focuses on the gene space and uses conserved sequence homology to genes of closely related plant species of smaller size and repeat content. Briefly, orthologous genes of multiple species are grouped and, for each gene family, one representative protein is selected thereby defining an orthologous group representative (OGR). Subsequently, based on conserved sequence homology, raw sequencing reads are associated to the OGRs. These associations define sequence read collections for each individual OGR. Each of these sequence bins is independently assembled using stringent parameters that can be estimated from *in silico* simulations of whole genome sequencing experiments. The assembled gene fragments are re-aligned and ordered along the OGR to facilitate estimation of the gene copy number of the target genome and further simplifies downstream analysis (Figure 1).

#### Definition of an orthologous gene set

In a first step, orthologous gene clusters were computed for the reference genomes of three grass genomes (*Brachypodium distachyon*[13], *Sorghum bicolor*[14] and *Oryza sativa*[15]) originating from different grass sub-families plus publicly- available barley full-length cDNAs. The OrthoMCL software version 1.4 [16] was used to calculate pairwise sequence similarities between all input protein sequences using BLASTP [17]. Markov clustering of the resulting similarity matrix defines the orthologous cluster structure.

A total of 86,944 coding sequences from these four grasses were clustered into 20,496 gene families. 9,843 clusters contained sequences from all four genomes.

In a second step, one representative gene model was selected from each of the 20,496 orthologous gene clusters, using the following strategy: 1. BLASTX [17] of all contigs from the bread wheat LCG assembly against all grass genes used in the OrthoMCL analysis; 2. Select the gene in each cluster that concentrates the most distinct wheat contigs; 3. If genes pool the same number of wheat contigs, select the one with the longest protein sequence as the representative gene model.

Genes with associated wheat contigs were identified for all but 445 gene clusters resulting in a total of 20,051 representative gene models.

#### Allocating the sequencing reads to orthologous gene representatives

The 454 sequence reads are clustered to the orthologous gene representatives using conserved sequence similarity. Thereby, pre-processing of the raw 454 sequencing data is a helpful approach to reduce computational complexity. Especially repetitive sequences, which represent up to 80% of the grass genomes [18,19], considerably extend search space, and thus memory as well as time requirements. Moreover, increased gene copy numbers caused by repetitive mechanisms and transposable element activity complicate and hampers downstream gene family analysis. For a fivefold whole genome shotgun dataset from hexaploid wheat using this strategy, 77% of raw sequence data was removed and 24 Gb out of 83 Gb of sequence kept for the orthologous group assemblies.

Afterwards reads were aligned to the orthologous gene representatives using BLASTX [17] and reported alignments filtered for minimum alignment length (minimum of 30 amino acids (AA)) and minimum alignment identity. Thereby, different alignment identity thresholds were applied accounting for the different evolutionary distances between bread wheat and the reference species used for the orthologous group analysis. Overall, 4 million (6%) of the wheat repeat-filtered sequencing reads passed the applied alignment criteria, and approximately two-third (68%) of these matched a single OGR. For the remaining 454 reads, that map multiple representatives, only the first-best match (FBH), were considered. Almost all (19,483) of the selected orthologous representatives were detected by at least one 454 sequence read indicating the good representation of the majority of wheat genes in the whole genome shotgun sequence dataset.

#### Generating gene-centric “sub-assemblies” using the Newbler overlap assembler

Sequence information and quality scores were extracted for the 4 million aligned 454 reads from the original sequencing library files. For each orthologous group, individual assemblies were computed using the Newbler de novo assembly software, which generates larger contigs based on overlaps between reads [20]. Thereby, the assembly parameter specifying the minimum alignment identity (*mi*) to accept overlaps between reads has to be selected with caution. On the one hand, a too relaxed *mi* parameter may cause a collapse of homeologous sequence copies, but, on the other hand, too stringent *mi* parameter would overestimate gene copies due to sequencing errors. Two methods applied for selection of the best *mi* parameter are discussed in the following section. The final set of “sub-assembly” sequences was created by combining singletons (454 reads that do not overlap with any other reads) and the assembled contigs (> = 100 bp).

**Figure 2:**
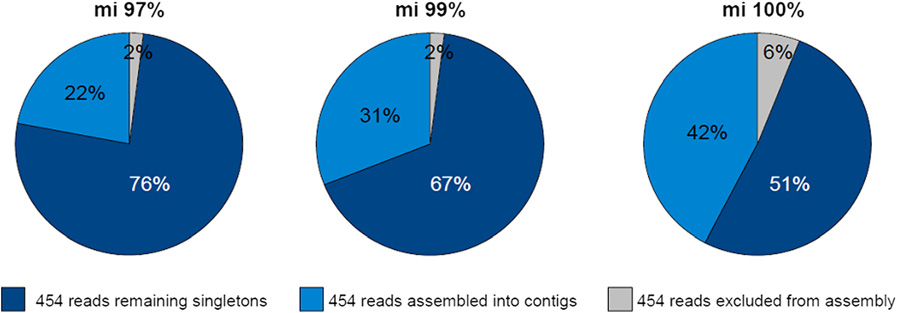
**Newbler assembly statistics of 454 reads for different minimum overlap identity parameters.** Three orthologous assemblies were performed using 97% minimum alignment identity (*mi*), 99% and 100%. For each assembly, the number of 454 remaining singletons (no significant overlap to any other 454 read), the number of assembled 454 reads and the number of excluded 454 reads (ultra-short reads, outlier reads, repeat reads) were counted.

The assembly results are strongly dependent on the chosen minimum alignment identity parameter. Whereas, 76% of reads were assembled into contigs applying 97% minimum alignment identity, the number of assembled reads dropped to 51% applying a minimum alignment identity of 100%, respectively (Figure 2). On the contrary, the number of 454 reads remaining singletons almost doubles between 97% and 100% *mi*. This fact already indicates that small-scale adjustments of this parameters influence the assembly results and the subsequent downstream analysis. Since this parameter is thus of critical importance in the following we discuss two method for the selection of the minimum overlap identity parameter in the following sections.

#### Estimation of gene copy number based on gene coverage

**Figure 3:**
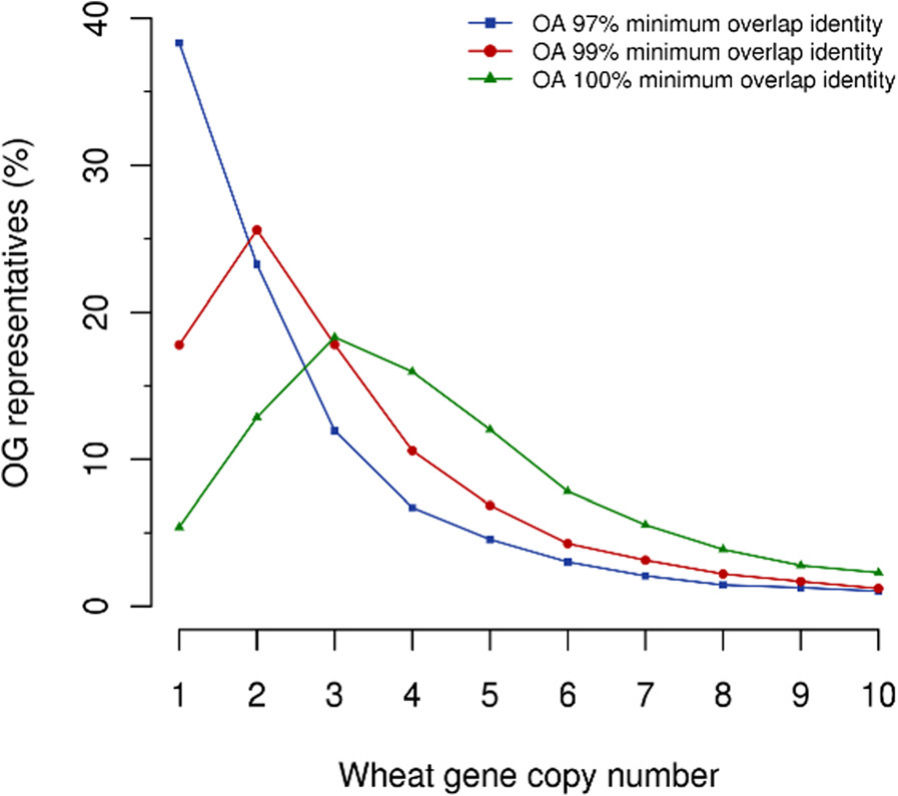
Wheat gene copy number in dependency of the applied assembly minimum alignment parameter.

In order to estimate the gene copy number, the orthologous sub-assemblies were re-aligned to the corresponding template gene. The same alignment parameters used for mapping the raw sequencing reads were applied. Thereby, spliced alignments of sub-assemblies were additionally considered and all consecutive high-scoring segment pairs (HSPs) along the template protein sequence accepted. Using this strategy, we account stretches of non-coding sequence regions of the sub-assemblies which span and/or reach into introns or 5′ and 3′ UTRs. Then, the alignments of the sub-assemblies were transferred into a position-specific hit count profile by counting the number of aligned sub-assemblies located at a specific amino acid position of the template sequence. The algorithm converts the hit count profiles into a cumulative coverage distribution. The distribution curve ranges from 1 to the maximum hit-count in the profile by only considering template positions that are tagged by one or more sub-assemblies. Based on that profile, the gene copy number is determined as the hit count assigned to C% of the OG representatives, whereby C is defined as the minimum fraction of the covered template. As already indicated above, the estimated gene copy number is dependent on the assembly parameters. As shown in Figure 3, the more stringent the *mi* parameter was chosen, the higher the estimated gene copy number.

#### Estimation of assembly parameters and evaluation of the copy number predictions

As already shown in Figure 2 and Figure 3, the correct selection of the Newbler parameter –*mi*, which specifies the minimum identity in order to combine two reads during the assembly, is a major factor affecting both the assembly outcome and the downstream gene family analysis based on predicted gene copy numbers. To estimate the correct Newbler parameterisation two simulation experiments were applied: (i) simulation of an whole-genome sequencing experiment for a diploid reference genome of similar genome size and repeat and gene composition and (ii) an *in silico* generation of an hexaploid gene set with similar sequence differences as in the homeologous genes of the target species.

#### Simulation of a whole genome sequencing experiment

Established reference assemblies or complete genome sequences of a reference species with similar genome size and structure can be used for the *in silico* simulation reflecting the experimental setting as achieved for the target genome sequence. Thereby, genomes with different degree of polyploidy could be used for this evaluation by adjusting the expectation of the reference gene family size. For the bread wheat genome analysis, the repeat masked genome sequence of maize (ZMb73, version 5b.60; http://www.maizegdb.org) was used and first, the “real” gene family size (gene count) identified by OrthoMCL clustering of the 39,656 annotated maize proteins and the reference protein data sets. However, because highly similar gene sequences, which could not be discriminated with the applied identity cut-offs during the sequence assembly, would artificially lead to significant underestimation of predicted gene counts, CD-Hit [21] nucleotide similarity clustering was performed at different similarity levels (97%, 99% and 100%, respectively) and all but one of the redundant sequences was retained. We selected more than 15,000 OrthoMCL cluster including one or more maize genes as well as exactly one OGR. Thus, counting the number of grouped maize proteins provided reference values for subsequent analysis and comparisons.

Next, the input data set for the orthologous assembly was designed and a collection of raw 454 sequencing reads created from the maize reference genome assembly applying with 5× genome coverage along with a uniform error rate of 0.5% [22]. Then, the above described protocol for the orthologous assembly was carried out with varying minimum overlap identity between 97% mi and 100% *mi*, respectively, and the gene copy number was predicted for the orthologous assembly.

#### Simulation of a polyploidy gene catalogue

Complementary to the above described evaluation method, a second evaluation was implemented, which in addition to the gene count simulates the polyploid effect size in the whole genome sequencing experiment. For this purpose, the transcript sequences of the 28,236 rice gene models (RAP2 annotation), including coding sequences as well as non-coding 3′ and 5′ UTRs and introns, were aligned against the orthologous gene representatives. Thereby the gene copy number in the rice data set was determined for each OG representative. Then, the aligned rice transcripts were triplicated and random single nucleotide variants (SNVs) were introduced to simulate the sequence similarity of the homeologous genes in the polyploid target genome. For example, for bread wheat nucleotides were randomly exchanged with p = 0.01 (1 nucleotide change per 100 bp) in coding transcript sequences and p = 0.04 (4 nucleotide exchanges per 100 bp) in non-coding transcript sequences, respectively. Then, 454-like shotgun reads were simulated (5× genome coverage), re-mapped against their corresponding OG representatives, sub-assembled with varying minimum overlap identity (97% mi, 99% mi and 100% mi) and, finally, the gene copy number predicted.

As expected, depending on the required stringency for read overlaps, the copy number prediction largely differs. In both evaluation methods 454 reads of different gene copy were collapsed by using 97% *mi*, whereas, requiring perfect alignment overlaps (100% *mi*) clearly results in an overestimation of the gene family sizes. As expected, we observed nearly perfect agreement of the expected 1:1 relationship between the expected and observed gene family size in the maize simulation, as well as the 1:3 relationship in the polyploidy simulation by using 99% alignment identity. This parameterisation allows compensating for sequencing errors, by simultaneously maintaining distinct gene copies that share high sequence similarity in coding regions. However, highly similar gene copies from large gene families may still collapse into single assemblies resulting in slight overall gene number underestimates.

### Sub-genome classification of bread wheat transcripts using machine learning

Besides sheer size and high repeat content hexaploidy makes the genome of bread wheat extremely challenging to analyse. Being able to differentiate between homologous genes of the three wheat sub-genomes (*A*, *B* and *D*) is of high importance not only for marker design and breeding but also to address open questions in the evolution and domestication of bread wheat.

Recent NGS sequencing approaches of the bread wheat genome generated a 5-fold 454 survey sequence, without being able to separate A, B and D sub-genome directly [9]. In principle, this can be facilitated applying a chromosome sorting technology [23], however, sorted sequences were only available for wheat linkage group 1 at this time [4]*.*

As a consequence, an alternative approach was established to classify wheat sub-assembly sequences for the A, B and D sub-genomes. Wheat sub-assemblies were generated by a stringent assembly of reads mapped to representative (for orthologous groups defined by OrthoMCL [16]) genes from the reference organisms *Brachypodium distachyon*[13]*, Hordeum vulgare, Oryza sativa*[15]*and Sorghum bicolor*[14] as well as the genome sequences of the D genome donor species *Ae. tauschii*[24], and the A genome relative *Triticum monococcum* (NCBI archive SRP004490.3), and cDNA sequence assemblies from *Ae. speltoides* (Trick&Bancroft, unpublished data) a member of the Sitopsis section to which the putative B genome donor belongs. Expecting that A- related sub-assemblies are more related to *T. monococcum* sequences, D- related sub-assemblies to *Ae. tauschii*, and B-related sub-assemblies to *Ae. speltoides,* sequence similarities of the sub-assemblies to each of these datasets would define and discriminate their origin.

In a first step, sequence similarities of each sub-assembly sequence to the wheat progenitor sequences were computed using BLAST [17]. Only sub-assemblies with BLAST hits to all three wheat progenitor sequences were considered for classification. Although a classification into A, B or D sub-genome derived transcripts seemed possible for many sub-assemblies by applying simple similarity cut-offs or rules, fixed similarity cut-offs appeared not suitable to separate the majority of sub-assemblies with confidence [9].

Consequently, several machine learning approaches were applied to the similarity matrix and evaluated for their performance. A major prerequisite represents the identification of a suitable training and test data set. We made use of wheat group 1 chromosome sequences which were separated into their sub-genomes (A, B and D) using flow-sorted chromosomes [4]. We extracted all wheat sub-assemblies associated with wheat group 1 chromosome sequences and classified them into A, B or D depending on their best hit. Every sub-genome classification was then complemented with the sub-assemblies’ similarities to *T. monococcum*, *Ae. speltoides* and *Ae. tauschii* sequences to create a training set compatible to all non-chr1 related sequences.

We applied a number of machine learning algorithms (e.g. Logistic Regression, Naive Bayes, Decision Trees and Support Vector Machine algorithms) from the WEKA package [25] (http://www.cs.waikato.ac.nz/ml/weka/) to this training set and evaluated the results by stratified k-fold cross-validation. The best compromise between precision and recall was observed for the Support Vector Machine algorithm (libSVM). This trained libSVM classifier was used to classify the set of genic wheat sub-assemblies into A-, B- or D-related sequences. The results of this classification are summarized in Table 1. Below defined libSVM probability estimate thresholds the classification was considered unreliable (e.g. in cases where a sub-assembly sequence matches equally well to all three progenitor sequence sets).

**Table 1:** Support Vector Machine classification results on wheat genic sub-assemblies

**Classification category**	**Wheat sub-assembly sequences with intact open reading frame**
**Used for classification**	**462,803 (100%)**
**Classified as A**	**94,949 (20.5%)**
**Classified as D**	**113,065 (24.4%)**
**Classified as B**	**97,923 (21.2%)**
**Not classified**	**157,166 (33.9%)**

With the recently published draft genome sequences of *Aegilops tauschii*[26] and *Triticum urartu*[27] two additional WGS datasets from wheat progenitors have since become available. As *T.urartu* is thought to represent the wheat A sub-genome progenitor even better than *T. monococcum*[28], using these datasets in an analysis update could potentially further improve the separation of sub-assemblies generated from the A sub-genome and therefore re-fine the overall result.

An alternative approach was taken in the separation of homeologs in the tetraploid wheat (AABB; “pasta wheat”) transcriptome, published by Krasileva et al. [29]. Here, a post-assembly pipeline including polymorphism identification, phasing of SNPs, read sorting and re-assembly of phased reads was used to separate homeologs.

### Physical, genetic and functional assembly of the barley genome

Barley is diploid and diverged from wheat approximately 12 million years ago (Mya) [30]. Barley is the first cereal genome where an anchored physical map has been reported on the basis of the entire genome. The physical map was constructed by combining different clone libraries using FPC [31] to assemble clones into contigs.

The genome size of 5 Gb made barley the largest genome for which a physical map has been constructed. 570,000 BAC clones were assembled into 9,265 fingerprinted contigs (contigs). Several clone libraries were combined to prevent that genomic regions would remain unrepresented. End-merging of contigs allowed to bridge overlapping contigs together, possible when marker evidences on both ends would indicate a merge. A wealth of different marker resources mostly linked to transcripts [32] but also by using *genotyping by sequencing* (GBS) technology helped to assign a majority of contigs to a genetic position. To increase the robustness of the resulting map, experimental markers were included. Apart from the construction of the physical map three different barley cultivars were sequenced under high coverage resulting in *whole genome shotgun* (WGS) assembly with approximately 2 gigabases (Gb) each. These WGS contigs, apart from forming the basis to derive gene models also helped to extend sequences on the physical map by extending clone end sequences. The WGS contig decorated physical map was then taken to infer a genetic position for physical contigs. Altogether 4 Gb out of the 5 Gb cumulative contig map length was anchored.

The anchoring and ordering of FPC and sequence contigs along the barley genome demonstrates that GBS technology combined with a transcript derived map is powerful to develop a rich and deep physical map even for the complex and large barley genome. Along with the annotation of genes it describes the functional and physical assembly of a cereal genome.

### The GenomeZipper approach

The GenomeZipper (GZ) [11,12] is a synteny driven approach to order and structure NGS survey sequences of grass genomes that lack a physical map. The approach can be applied to a variety of different data sets, i.e. 454 reads, contigs, or scaffolds grouped in individual chromosomes or chromosome arms. The approach exploits the widely conserved synteny among grasses [33] and uses corresponding syntenic intervals, as defined by heterologous, corresponding marker intervals among the species under investigation and suitable reference genomes to deduce a tentative ordering of genes in the corresponding regions. This approach can be undertaken for smaller regions of interest, whole chromosome arms and chromosomes on a genome wide level. The outcome is a virtual gene order map which integrates gene-based marker maps as well as conserved syntenic information from at least one sequenced model grass genome and NGS data. Initially applied on the barley genome [11,12], the genome zipper approach has now also been used with other grass species, such as wheat chromosome 4A [34], rye chromosome B [35] and Lolium [36].

**Figure 4:**
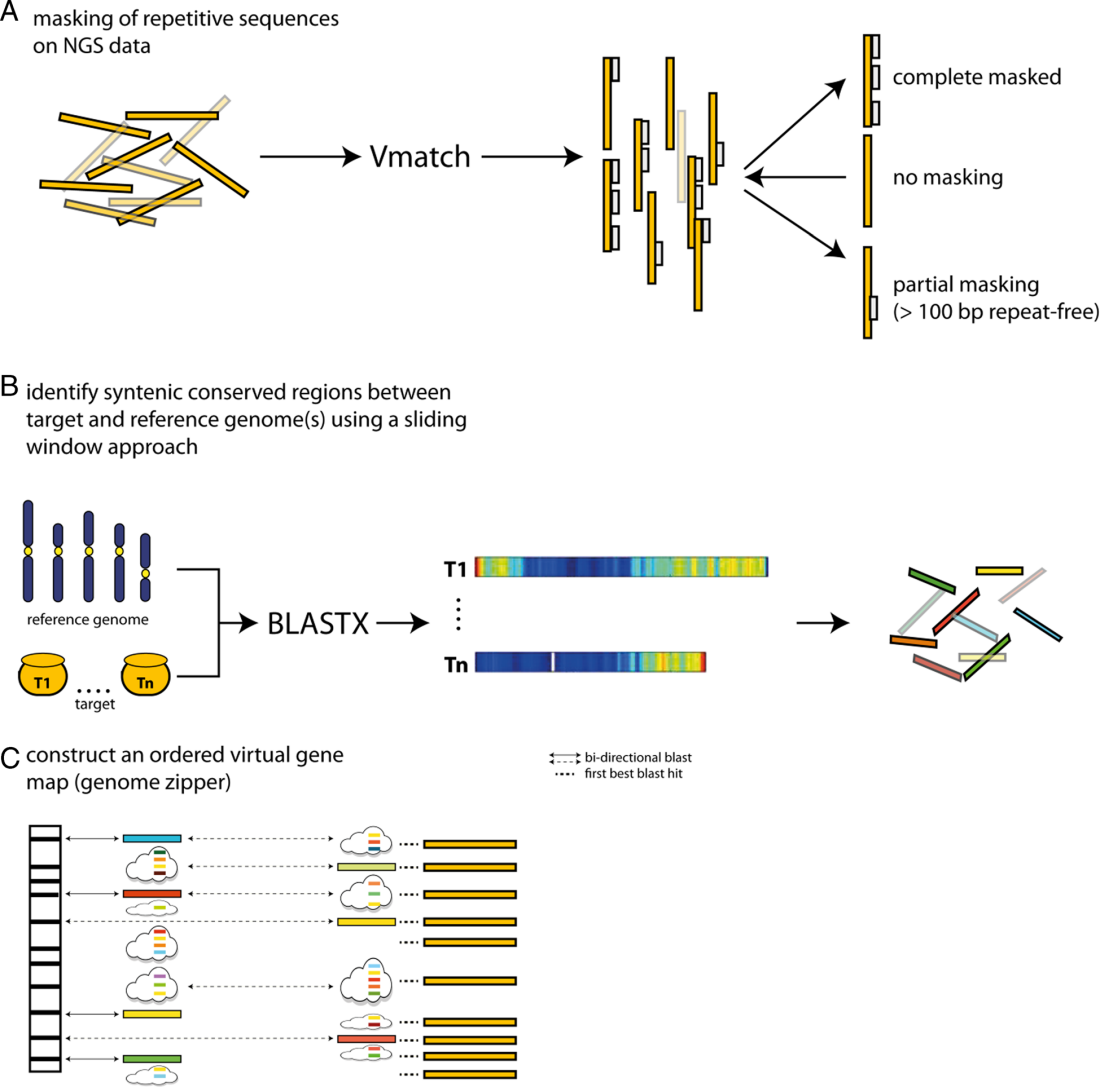
**GenomeZipper workflow.** The GenomeZipper approach can be divided into three individual steps which can be run independently: repeat masking **(A)**, detection of syntenic conserved blocks **(B)** and the ‘zipping’/integration **(C)** of all data sets into a virtual linear gene order model. **A)** The repetitive sequences (grey boxes) were filtered out from the NGS data (orange boxes) using Vmatch and the MIPS REdat Poaceae v9.0 library. Only sequences without repetitive elements or with at least 100 base pairs repeat-free regions were considered for the next analysis steps. **B)** The syntenic conserved regions between target and reference genome(s) were determined using BLASTX and a sliding window approach. The sequences were aligned against the reference genome(s) and the highly conserved genes (coloured boxes) were extracted and used for the construction of the gene map. **C)** The virtual gene map is constructed by integrating the syntenic conserved genes of one or multiple reference genomes and the NGS survey sequences along a backbone build by a genetic marker map of the same organisms as the target or a very closely related one. In a first step all conserved genes with a bi-directional blast hit to the gene-based marker are integrated into the zipper backbone. The remaining conserved genes (depicted in the clouds) are indirectly incorporated using the order deduced from the corresponding reference genome. The NGS data (orange boxes) are anchored to the ordered map by first best blast hits.

The GenomeZipper approach consists of three discrete steps: repeat filtering, detection of syntenic conserved regions and an integration step which provides an anchored, information-rich scaffold (Figure 4).

In the first step, filtering repeat regions out of the query sequence reduces computational effort in gene space estimation. In barley and wheat the repetitive amount was identified by aligning the 454 reads against the MIPS REdat Poaceae repeat library using Vmatch (http://vmatch.de).

In the next step, the conserved homologs between query sequence and one or multiple reference genomes are determined. For barley, three model grass genomes - *Brachypodium distachyon*[13], rice [15] and *Sorghum bicolor*[14] - were used to identify homologous regions. The sequence comparisons were done using BLASTX and only first best hits with at least 75%/70% sequence identity and a minimal alignment length of 30 amino acids were considered. The syntenic conserved regions are defined by the density of homologous matches between query and reference genome using a sliding window approach.

During the last step, the NGS data is structured and ordered using a high resolution genetic map and orthologous genes obtained in the previous step. Thereby the intervals as defined by genetically ordered markers are used as a scaffold to project the likely order of corresponding genes in these intervals. Gene order in these intervals is deduced from the order found in the respective reference genomes, whereby evolutionary closest reference genomes get highest rank. Once ordered, additional evidences, such as full length cDNAs and/or ESTs can be attached to the ordered gene scaffold.

The linear ordered gene maps provide a valuable resource for a variety of applications: (i) for marker development and to assist positional cloning [37], (ii) for comparative analyses of the conserved gene space [4], and (iii) to resolve the structure of a genome/chromosome and to establish the colinearity between grass genomes[34,35].

### Conclusions

Next generation sequencing technologies now start to enable to decipher large plant genomes such as those from many *Triticeae* (wheat, barley, rye) which until recently were difficult to access due to severe technological and economic restrictions. The assembly and analysis of these complex genomes remains a challenge and requires novel concepts and strategies. Here, we outlined and described a number of these concepts, that were developed and used to analyse and order genes from the recently published genome sequences of barley and hexaploid wheat. For barley, a physical and genetic map integration approach allows to positionally anchor ~21,000 genes. A complimentary approach, the GenomeZipper concept, makes use of the conserved gene order between grass reference genomes and many monocot crop genomes to anchor and order genes by an *in silico* approach in the complex wheat and barley genomes.

The hexaploid nature with three highly homologous sub-genomes makes the genome of bread wheat extremely challenging to assemble and analyse. To access the gene inventory, a set of orthologous representative genes was constructed from related and finished reference grass genomes. Wheat NGS reads from a 454 5× whole genome sequence survey were mapped onto these orthologous representatives and separately assembled in a stringent way to avoid collapsing of homologous (sub-genome derived) genes. Genic sub-assembly sequences were subsequently classified into A, B or D- sub-genome derived with a machine-learning assisted approach making use of differing sequence similarities to the A, B and D sub-genomes progenitor species.

The approaches and concepts outlined here may be readily applied to other complex genomes, even beyond plants, where direct sequence assembly and analysis is hampered by size and/or polyploidy but related, less complex reference genomes are available.

## Competing interests

The authors declare that they have no competing interest.

## Authors’ contributions

MS wrote the introduction, conclusions, and contributed to the wheat sections. MM contributed to the GenomeZipper section. MP contributed to the wheat sections and TN contributed to the barley section. KM supervised the manuscript. All authors read and approved the final manuscript.
